# Improvement in hearing after chiropractic care: a case series

**DOI:** 10.1186/1746-1340-14-2

**Published:** 2006-01-19

**Authors:** Joseph O Di Duro

**Affiliations:** 1 Palmer Center for Chiropractic Research, 741 Brady Street, Davenport, IA 52803-5287

## Abstract

**Background:**

The first chiropractic adjustment given in 1895 was reported to have cured deafness. This study examined the effects of a single, initial chiropractic visit on the central nervous system by documenting clinical changes of audiometry in patients after chiropractic care.

**Case presentation:**

Fifteen patients are presented (9 male, 6 female) with a mean age of 54.3 (range 34–71). A Welch Allyn AudioScope 3 was used to screen frequencies of 1000, 2000, 4000 and 500 Hz respectively at three standard decibel levels 20 decibels (dB), 25 dB and 40 dB, respectively, before and immediately after the first chiropractic intervention. Several criteria were used to determine hearing impairment. Ventry & Weinstein criteria of missing one or more tones in either ear at 40 dB and Speech-frequency criteria of missing one or more tones in either ear at 25 dB.

All patients were classified as hearing impaired though greater on the right. At 40 dB using the Ventry & Weinstein criteria, 6 had hearing restored, 7 improved and 2 had no change. At 25 dB using the Speech-frequency criteria, none were restored, 11 improved, 4 had no change and 3 missed a tone.

**Conclusion:**

A percentage of patients presenting to the chiropractor have a mild to moderate hearing loss, most notably in the right ear. The clinical progress documented in this report suggests that manipulation delivered to the neuromusculoskeletal system may create central plastic changes in the auditory system.

## Background

The broad category of hearing loss is the third most prevalent chronic condition in older Americans, following hypertension and arthritis [1]. Between 25% and 40% of the population aged 65 years or older is hearing impaired [2]. Hearing impairment refers to limitation of function or raised hearing threshold (inability to hear tones at a normal level) and this implies a total or partial loss of the ability to perceive acoustic information. The impairment may affect the full range of hearing or be limited to parts of the auditory spectrum. This impairment is expressed as decibels of hearing loss (dB HL) relative to the hearing of a normal population. The Veterans Health Administration has used the criteria of failure to hear a 40 decibel tone (40 dB threshold) as hearing loss, though other criteria can be used. Testing is also conducted at specific frequencies (250, 500, 1000, 2000 and 4000 Hz) as the ear is particularly sensitive to these signals which include the frequencies most important for speech processing.

The diminished ability to hear and to communicate is frustrating in and of itself, but the strong association hearing loss with depression and functional decline adds further to the burden on older individuals [3]. The onset of sensorineural loss, or presbycusis, is insidious and patients themselves are frequently unaware of their hearing loss. Hearing loss often goes undiagnosed because of its slow onset and the chiropractic patient population may be an ideal place for hearing screenings.

Chiropractic has long been associated with hearing. The first chiropractic adjustment given in 1895 was reported to have cured deafness. Wagner and Fend [4], from Germany, reported a case where a 36 year old male soccer player became suddenly deaf in his right ear with tinnitus following hitting the ball with his head. An audiogram showed that loss of hearing at 500 Hz and he was diagnosed by the physician as almost completely deaf in the right ear. Following adjustments to the thoracic spine, (T6) the right sacroiliac joint and restrictions on the right side of the neck (C2–C4) were adjusted, with an audible pop detected during the manipulation, the patient reported a sudden improvement of hearing in that he could hear a whisper from four meters. The post audiogram showed his hearing had returned.

Hulse [5], also from Germany, found subjective hearing disorders in 62 patients with palpation-defined cervical spine dysfunction. In 40% of these patients, an audiometric loss of frequency tones in the low frequency range (1000 Hz) was observed. Of the patients that were examined in his study, 68% presented with unilateral and consistently right-sided deficit in hearing. Hulse concluded that this form of hearing loss is reversible and that upper cervical chiropractic manipulation to the neck was his treatment of choice [5].

Svatko [6], from Russia, examined 105 patients for cervical spine pathologies and found that 19 of these patients showed bilateral hypoacusis, (hearing loss) though more severe on one side. Seventeen of these subjects' hearing improved and Svatko concluded that there was a potential to improve "dull" hearing by manual manipulation. His therapy of choice was chiropractic manipulation of the functional blocks in the upper (OCC-C1) cervical spine [6].

This current study examined hearing impairment in a chiropractic patient population and the effects of a single, initial chiropractic visit on changes in audiometry in these patients after chiropractic care.

## Case presentation

### Methods

A sample of convenience consisting of fifteen consenting patients (nine male, six female) with a mean age of 54 years (range 34–71), obtained from a panel of 200 patients presenting for chiropractic care in Vicenza, Italy during one year (June 2000 to June 2001) was the basis for this case series study. As seen in the patient characteristics in Table 1, no patients had a chief complaint of hearing loss or impairment. Audiometric screenings were performed on each new patient entering the clinic regardless of complaint (n = 200). A Welch Allyn hand-held AudioScope 3 was used to screen the speech frequencies (tones) of 1000 Hz initially, then 2000, 4000 Hz and finally 500 Hz at three different fixed decibel levels of 20 dB initially, then 25 dB and finally 40 dB. The tones were presented at random intervals for objectivity. The AudioScope has been shown to be a sensitive, valid and reliable testing tool for hearing loss which is quick and easy to use, well tolerated by patients and does not require a sound treated room [7-15]. The majority of this patient group (93.5%) could hear 11–12 total tones in each ear. Those patients selected for this study demonstrated a hearing impairment in which they failed to hear a significant number of the 12 possible tones in either ear, on this initial exam. They were re-evaluated immediately following their first chiropractic adjustment.

**Table 1:** Patient characteristics at baseline Audiometric exam.

		TOTAL TONES HEARD	
Gender/Age	Presenting Complaint	Right	Left
F/67	NP/HA/VERTIGO	1	5
F/65	NP	1	3
M/71	LBP	2	8
M/49	NP	2	7
M/34	LBP	3	5
F/61	NP/LBP	3	4
M/48	NP	3	4
M/37	LBP	3	8
M/38	LBP	4	7
M/57	LBP	4	6
M/71	VERTIGO/LBP	5	5
F/56	DEPRESSION	5	8
F/64	LBP	6	5
M/51	MIGRAINE	6	3
M/41	NP/LBP/SH	7	5

		**55**	**83**

NP = neck pain; HA = Headache; LBP = low back pain; SH = shoulder

Examination and palpatory findings were used to define areas of joint dysfunction and each patient received a high velocity, low amplitude thrust in the thoracic, lumbar spine and locomotor system including extremities. No "specific" adjustment was given to solely restore hearing.

### Results

In the patient group with hearing impairment, the total number of tones heard on initial exam was fewer in the right ear (55 tones) than the left (83 tones). The normal patient group heard approximately 120 tones in each ear on the initial visit. After a single chiropractic intervention, the total tones heard increased to 104 on the right and 111 on the left (an increase of 49 and 28 respectively) (See table 2).

**Table 2:** Total tones heard (of possible 12) Pre versus Post.

	PRE		POST	
Gender/Age	Right	Left	Right	Left
F/67	1	5	7	8
F/65	1	3	6	6
M/71	2	8	6	6
M/49	2	7	8	8
M/34	3	5	6	8
F/61	3	4	5	5
M/48	3	4	11	8
M/37	3	8	8	10
M/38	4	7	8	10
M/57	4	6	8	9
M/71	5	5	6	4
F/56	5	8	5	5
F/64	6	5	4	7
M/51	6	3	8	10
M/41	7	5	8	7

**TOTAL**	**55**	**83**	**104**	**111**

Using the Ventry & Weinstein criteria [9-11,13,15] of hearing loss that considers missing 1 of 4 tones at 40 dB in either ear, on a Welch Allyn AudioScope 3, all of these patients were impaired. Post chiropractic intervention, 6 had their hearing restored, 7 had hearing improvements, 2 did not change and none worsened (see Table 3).

**Table 3:** Criteria of hearing loss missing one of eight tones (total of four possible in each ear) at 40 dB before and after one chiropractic manipulative visit. All patients classified as hearing impaired before.

	PRE		POST		CHANGE	
Gender/Age	Right	Left	Right	Left	Right	Left
F/67	1	4	3	4	+2	
F/65	2	2	2	3		+1
M/71	2	3	2	4		+1
M/49	2	4	4	4	+2*	
M/34	1	4	3	4	+2	
F/61	1	2	2	3	+1	+1
M/48	2	3	4	4	+2*	+1*
M/37	3	2	4	4	+1*	+2*
M/38	2	4	4	4	+2*	
M/57	2	3	4	4	+2*	+1*
M/71	3	3	3	3	NC	
F/56	2	3	3	3	+1	
F/64	1	0	2	4	+1	+4
M/51	2	4	2	4	NC	
M/41	2	4	4	4	+2*	

**TOTAL**	**28**	**45**	**46**	**56**	**+18**	**+11**

* = hearing restored; NC = No Change

Using the Speech-frequency criteria of hearing loss that considers missing 1 of 8 tones at 25 dB in either ear using a Welch Allyn AudioScope 3 [7,8,12,14,15], all of these patients were hearing impaired. Post chiropractic intervention none of these patients had their hearing completely restored but 11 improved, 3 patients in both ears, while 4 showed no change and 3 patients missed an additional tone (see Table 4).

**Table 4:** Criteria of hearing loss missing one of eight tones at 25 dB (total of four possible in each ear) before and after one chiropractic manipulative visit. All patients classified as hearing impaired before.

	PRE		POST		CHANGE	
Gender/Age	Right	Left	Right	Left	Right	Left
F/67	0	1	2	4	+2	+3
F/65	2	2	2	2	NC	
M/71	0	1	0	1	NC	
M/49	2	2	2	2	NC	
M/34	1	2	2	2	+	
F/61	2	1	1	1	+	
M/48	2	1	3	2	+	+
M/37	2	2	2	2	NC	
M/38	1	2	2	3	+	+
M/57	0	1	2	2	+2	+1
M/71	2	0	2	1	+1	
F/56	0	1	1	1	+1	
F/64	0	0	1	2	+1	+2
M/51	2	1	2	2	+1	
M/41	1	2	2	2	+1	

**TOTAL**	**17**	**19**	**26**	**29**	**+9**	**+10**

NC = No Changep

### Discussion

The current observational study cannot prove a cause and effect relationship. The limitations to this current study are the small sample size and that there was no blinding of the investigator though patients were blinded to the fact that hearing would be tested post-chiropractic care. Furthermore, no true control group or randomization of testing sequence was employed and potential alternative explanations as to the natural history of hearing loss may explain our results, for example some learning effect of the test.

#### Possible mechanisms

The auditory system is inherently plastic, permitting us to learn to identify new voices, speak new languages and sing new songs. The rapid changes observed in our sample group were characteristic of those occurring in central adaptive mechanisms [16]. These central plastic changes are most likely the result of relatively simple alterations in the balance of excitatory and/or inhibitory inputs produced by manipulative care when examining central auditory processing.

#### Cortical mechanisms

Each primary sensory cortex, in this case the auditory and somatosensory, project to nearby higher order areas of sensory cortex, called unimodal association areas, that integrate afferent information for a single sensory modality [17]. The unimodal association areas in turn project to multimodal sensory association areas that integrate information about more than one sensory modality. Animal experiments indicate that dynamic cortical reorganization of the representation or tonotopic map of the cochlea, the primary organ for hearing, occurs when the cochlea is lesioned [16]. Specifically, cortical regions deprived of normal peripheral input show expanded representation of lesion-edge frequencies. Reorganization of cortical and behavioural activity associated with sensory deprivation has also been demonstrated in humans [16]. Therefore, it is possible that a long standing decrease in activation of the auditory cortex and primary association areas, which may occur in insidious hearing loss, could produce a central auditory processing disorder (CAPD) [18] and that, in turn, could serve to explain the areas of hearing loss and rapid restoration seen in our patient group.

The concept of central plasticity (i.e. the central nervous systems ability to adapt to environmental influences) presumes that changes in one sensory modality may create a convergence upon other areas of the cortex that integrate that information into a polysensory event. Some authors have pointed to the site of this neuronal plasticity as characteristic of the non-primary auditory thalamus and cortex [18]. Cortical integrity relating to task-conditioned speech sounds is reflected in lateralized supratemporal cortical responses possibly in concordance with the left hemispheric dominance in language [19]. A certain level of left/right dissociation in the processing of tones within the speech sound range may be reflected in the significantly greater unilateral hearing loss which we recorded in the right ear. If this is the case, then the changes induced by chiropractic evoked somatosensory potentials via physical adjustments create changes in both hemispheres as indicated by our data. We noted that despite generalized and predominantly right-sided deficit detected in the audiograms of each patient, the total number of tones recognised post chiropractic care surprisingly became evenly distributed and symmetrical (Table 2). This may a global change in neural activation rather than a change in one specific modality.

#### Thalamic mechanisms

Recent electrophysiological evidence has changed the traditional view that language and memory being primarily in the cortex to focus on the role of subcortical structures [20]. Loss of language function in a patient after a focal infarct of the left ventral lateral thalamic nucleus extending to the anterior part of the pulvinar [21] exemplified the way the left thalamus brings online the cortical networks involved in language processing. This form of "selectively engaging" positioned the thalamus as integral in activating post-synaptic areas [22]. This concept places the thalamus as an alerting system activating a mosaic of specific discrete cortical areas appropriate to a particular task and maintaining other cortical areas in a state of relative disengagement (inhibition). Asymmetric hemispheric responses to speech sounds are well documented, however thalamic as well as cortical specialisation to language has also been demonstrated, the left being more involved [20]. New evidence derived from a battery of studies on patients undergoing stereotatic thalamic operations for the treatment of chronic pain, dyskinesias, (Parkinsonism) dystonia and tremor demonstrated that when the ventral lateral thalamus, long considered the "motor" area of the thalamus, was stimulated on the left, performance on tests involving simple speech sound was enhanced. However, when lesions were administered to the left thalamus, dichotic listening performance was impaired [23]. The results suggest that the thalamus is involved in generating a "specific alerting response" that acts as a gating mechanism which controls the input and retrieval of specific items [23]. Specifically, activation of the reticular nucleus of the thalamus changes an "arousal threshold", thereby affecting language processing and learning. As an integrating group of neurons that connect to every level of brain tissue, it appears that the left thalamus plays a central role in manifesting arousal control and contributing to excitation or inhibition of the auditory system.

In a study of 500 participants, Carrick [24] examined the central effects of cervical spinal manipulation on the changes in dimensions of the visual field's blind spot. His results suggest that cervical manipulation has a strong significant ability to change and increase contralateral thalamic and cortical activity. Carrick postulated that changes in amplitude of muscle stretch receptors and joint mechanoreceptors from manipulation change the amplitude of somatosensory receptor potentials, which in turn, influence the frequency of firing of cerebello-thalamocortical loops responsible for maintaining a central integrated state of the cortex [24].

#### Brainstem mechanism

The changes in a persons' ability to hear tones at speech threshold would fall under the classification of central adaptive changes or plasticity. There is no doubt that central plastic changes occur in the brainstem, specifically at the level of the vestibular nerve. Central plastic changes and recovery in vestibular nuclei adapt so rapidly that complete unilateral labyrinthectomy (complete damage to one labyrinth) should create extreme vertigo and imbalance. However, patients can become asymptomatic in less than two weeks [25]. Spontaneous regeneration and recovery of hearing function of central auditory pathways after transection of the ventral cochlear tract in the pons have been noted in young rats [26]. Plastic changes in the auditory system have been noted to take place much faster in central systems than in peripheral system following a reversible cochlear damage (the primary receptor for hearing) [27]. In an animal model, employing similar frequencies and decibels to those in our study, an acid was administered at the inner hair cells (the location of the auditory nerve synapse) in the cochlea. This excitotoxic damage is reversible and in time hearing was restored. The investigators discovered that the inferior colliculus evoked potential (IC-EVP) was restored much more rapidly than the compound action potential (CAP overall) measured for the auditory nerve. This restoration was so fast that the IC-EVP was restored to nearly 80% of baseline at between one to five days, while the CAP overall remained below baseline even at 30 days. Furthermore, the CAP amplitudes remained depressed while the IC-EVP amplitudes tended to overshoot their baseline values by some 20% [27]. In other words, when the threshold for hearing was compared, no difference could be discerned between the response threshold from peripheral and central measurements, though the synaptic areas did not contribute equally to these the adaptive or plastic changes. This research offers a new perspective on central plasticity and it is important to note that these rapid changes were measured at the level of the inferior colliculus (IC) does not mean that the IC is the site of plastic change. It may be the case that functional and possibly structural changes have occurred at lower levels of the brainstem and are merely being reflected "upstream" in the response of neurons in the IC.

Another possible site for confluence of somatic and acoustic input is the vestibulo-cochlear system within the brain stem. Unilateral hearing loss is frequently noted in persons with vertigo [28-30]. In fact, between 8% to 44% of vertigo cases are associated with a chronic ipsilateral sensorineural hearing loss [28]. The vestibular nuclei integrate signals from the vestibular organs and visual system with that of the somatic system. Therefore, it is possible that changes in the vestibulo-cochlear system of the brainstem brought about through afferent information of somatic structures affected by chiropractic adjustments may influence the integrity acoustic processing and hearing.

## Conclusion

A percentage of patients seeking chiropractic care have a mild to moderate hearing loss, identified by audiometry. In accordance with other reports, the clinical progress documented here suggests chiropractic care may benefit hearing loss and that chiropractic adjustments to various areas of the spinal column and locomotor system may have an effect on central auditory processing, though alternative explanations can not be disregarded. There is a difference in the unilateral aspect of the hearing deficit noted in the right ear of patients in this current study as reported in others. The observations documented in this case series provide limited support to previous works indicating that, when hearing is tested immediately after a single chiropractic adjusting visit, hearing may be improved in both ears. Further research in this area is required, in the form of a well designed randomised controlled trial.

## Competing interests

The author, Joseph O Di Duro declares no competing interests, financial or non-financial.
